# Discourses in Subjective Experience on Rehabilitation

**DOI:** 10.1007/s42087-021-00229-8

**Published:** 2021-06-01

**Authors:** Sirpa Ylimaula, Teemu Suorsa

**Affiliations:** 1 Faculty of Education, Sociology, University of Oulu, Oulu, Finland; 2 Research Unit of Psychology, Faculty of Education, University of Oulu, Oulu, Finland

**Keywords:** Discourses, Individualisation, Subjectivity, Unemployed young people, Vulnerability

## Abstract

In Finland, youth workshops support participants’ everyday management, social empowerment and employability skills, facilitating their access to education and work. In this study, we analysed conversations with ten participants about their experiences in non-formal learning workshops in Northern Finland. We explore how participants described personal transformation borne by workshop participation and how the prevalent discourses on youth (un)employment manifest in participants’ reasons for action. In the analysis, we identified (1) how participants assessed their situations; (2) their thoughts, feelings and actions in relation to their situations; and (3) their grounds for these thoughts, feelings and actions. The societal dimension of subjective experience is articulated by identifying the prevalent societal discourses featured in participants’ descriptions. The analysis showed that participants described themselves as vulnerable prior to the intervention and motivated after the intervention. Participants experienced tailored support as being significant in fostering the change. Individual descriptions of how the learning and training conditions were experienced constitute valuable knowledge about the possible ways of acting and experiencing in relation to common structures. With the help of these descriptions, it would be valuable for researchers and participants to continue the conversation about the discourses to enhance the participants’ conscious participation in maintaining and changing their living conditions.

## Introduction

The situations of socially excluded young people are neither homogeneous nor stable (Aaltonen et al., 2015) and—in the case of young people without post-primary education—overlapping social risks include unemployment, poverty and the need for rehabilitation (Hiilamo et al., 2017). Large groups of young people need to find alternative ways to education or the labour market, but transitions from school to work can be traumatic and challenging (Eurofound, 2016; European Union, 2020; Paving the Way, 2017). Labour market citizenship is often seen as the most important form of citizenship, and those outside the work force are a problem (Aaltonen et al., 2015). Early school leaving is costly for individuals, society and the economy (Borbely-Pecze & Hutchinson, 2014). The European Union (2020) has recently recommended strengthening the Youth Guarantee in response to the economic downturn caused by the COVID-19 pandemic. Previous recessions show that young people suffer more than older people, and therefore, all young people under the age of 29 should receive an offer of employment, education, apprenticeships or traineeships within 4 months. The recommendation calls for a stronger focus on vulnerable groups, the development of individual action plans, a more holistic approach to counselling and guidance and the validation of non-formal and formal learning outcomes. According to Borbely-Pecze and Hutchinson (2014), European politics emphasizes that strong personal commitment of learners is a preventive step against early school leaving. Work-based learning provides young people with knowledge of what occupational areas they are interested in and training that builds specific competences needed for jobs. Work-based learning is part of lifelong learning and includes formal and non-formal learning. For years, and in several countries in Europe, production schools and youth workshops for unemployed youth have provided opportunities for non-formal learning which occurs through activities designed, but not necessarily intended, for the purposes of learning (Paving the Way, 2017). Participants gain new skills through concrete tasks and learn to recognize their strengths through feedback, and individual coaching supports the increase in both professional skills and social empowerment. The documentation and assessment of learning are tools to motivate participants to seek further education.

Examining education-work transitions is challenging, as transitions occur at both the macro and micro levels (Raffe, 2014). Changes in transition systems in response to global pressures and several trends have had been observed in all countries: entry into the full-time labour market is delayed; young people are disadvantaged in the labour market; and transitions have become longer, more differentiated and less predictable.

Inequalities in the transition system are intertwined with and shaped by social differences, such as gender, social class, ethnicity, nationality and family relations. Society has always been in an ambiguous relationship with individual autonomy (Bauman, 2012). It has been its enemy and has been a prerequisite. Only the proportion of threads and chances of this ambivalent relationship have changed in the course of modern history. Currently, discourses that redefine inequalities as individualised risks and understand social problems in light of psychological dispositions are taking over (Thompson, 2011). Young people face more complex opportunities and are forced to navigate themselves to construct a marketable self. Low attainment levels and negative attitudes and behaviours are now seen as characteristics of young people, families and communities rather than as consequences of inequality. Transitions always take place within a context that shapes experience, and outcomes are always dependent on resources, and as a result, the relationship between context and subjectivities is a powerful tool for understanding the impact of social policies on people’s lives (Furlong et al., 2011). Thus, since individualization is here to stay, there should be more debate and negotiation between the individual and the common, private and public good (Bauman, 2012).

Measures and programmes for young people are tailored to reintegrate them into society, but little research has considered how these recipients experience the services (Aaltonen & Kivijärvi, 2018). Changes in the conceptions of youth, services and labour markets have increased interest in encouraging young adults into these programmes. In macrolevel discussions, participants’ own views on the meaningfulness of the services might be elided, and studies of problems such as the lack of services do not combine individual experiences (at the micro level) with structural factors. It is, therefore, important to identify the services that young people consider functional and successful.

In Finland, in 2020, unemployed persons not in education, training or performing compulsory military services accounted for 10% of the group aged 15 to 29 (Statistics Finland, 2020). Young people still want to work, and people are seldom unemployed by choice. The Youth Barometer (Haikkola & Myllyniemi, 2020) has measured the attitudes of young people aged 15 to 19 in Finland since 1994, and for years, the barometer has shown the importance of work as life value for young people. Youth Barometer’s 2019 interview material, based on 1907 telephone interviews, 75% of people aged 15 to 29 said they would rather accept a job than live on social security. In the 2017 Youth Barometer, based on 1902 phone interviews, 17% of people aged 15 to 29 said that they had quit school and the most cited reason for dropping out was selecting the wrong field (Pekkarinen & Myllyniemi, 2018). There was also a strong appreciation for education among dropouts, as more than 90% believed education would improve their chances of employment and lack of support was the main reason young people ended up without education after quitting their studies. In Finland, structural inequality includes nonspecific learning disabilities, which have an impact on transitions to work or education (Eriksson, 2018). Nonspecific learning disabilities refer to cognitive impairments that are difficult to find or identify, and they hinder learning (Närhi et al., 2010). People with such disabilities are not included in any statistics, and since support is tied to medical diagnosis or lack of it, support remains a structural, regional and local problem, and as young people cannot influence care provision, the young person’s diagnosis—not the individual’s need for support—determines the support given (Eriksson, 2018). For many people with nonspecific learning disabilities, not receiving special support means that their chances of finding work will be minimal. The researcher emphasizes the importance and need for individualised support for young people with learning difficulties, especially in the transition from primary to vocational education and from vocational education to employment.

Society cannot be ignored in regard to individuality. Human beings do not simply live under conditions but are participating in producing the conditions under which they live (Holzkamp, 2013). Belonging to the work community is a key process in the development of human abilities, and the need for recognition is a prerequisite for the development of self-awareness. The question is as follows: can everyone develop their abilities and contribute to society, and if not, what are the impacts on the functioning of society (Miettinen, 2016)? School has a significant impact on recognition, and in the daily lived experience of education and schooling, some learn to labour (Willis, 1977), and others learn to take positions of power (Choudry & Williams, 2017). In general lines, we agree with Billmann (2019), who suggests that we should pay attention to both discourses (Foucault, 1980) and subjectivity (Holzkamp, 2013). In the following, we describe this conceptualization and explain how it frames our empirical research.

## Theoretical Framework and the Research Questions

Michel Foucault’s (1980) work offers a perspective through which to articulate lifelong learning and the wider means and effects of it that are embroiled with relations of power (Nicoll & Fejes, 2008). Central arguments for lifelong learning are a qualified workforce with necessary skills and an individual’s responsibility to learn. For Foucault, there is no general history of human subjectivity; instead, we should make visible the discursive conditions and techniques that shape such subjectivities (Nicoll & Fejes, 2008). In neoliberal politics, which emphasizes market efficiency, people are seen through an economic lens, with life becoming the capitalisation of the self (Ritzvi & Lingard, 2009). Young unemployed people are governed in several ways (Brunila & Ryynänen, 2017), and governmentality here represents an intermediary between subjectivity and power (Billmann, 2019).

In many countries, therapeutic ideas and implementations of education policy and teaching seem to extend their reach (Brunila & Ryynänen, 2017; Brunila et al., 2018). Training projects focus on shaping the self to increase employability. Unemployment is seen as a personal problem, not a structural problem; that is, a lack of employment is due to a lack of employability, which can be cured by becoming more willing to adapt. These approaches have a common focus: emotional and psychological vulnerability. Researchers are concerned that therapeutic interventions are replacing education and individualising social problems. According to them, we should be more concerned with how the employability of young people is obtained. The discourses on youth transitions might influence the individual’s sense of self, and perhaps, we should examine school-to-work transitions in political, practical and discursive terms, as they might shape young people’s intentions and possibilities (Brunila & Lundahl, 2020).

Foucault’s approach, however, can be said to undermine the idea of autonomous agency (Rey, 2019). In this article, we adopt a subject-scientific approach that accentuates the societal nature of human beings and action. Furthermore, the subject-scientific approach accentuates sociomaterial practices and conduct of everyday living (Højholt & Schraube, 2016; Holzkamp, 2013; see also, e.g., Rey, 2019). The concept of conducting everyday life has opened a special approach to understanding the relationship between the individual and society. This concept goes beyond the perspective of autonomous individuals and recognizes that individual life is less determined by humans themselves than they would like to think (Højholt & Schraube, 2016). One’s subjective situation is a personal assessment of the actual possibilities of living and acting in the available action environment and reasons for action can only be found from the standpoint of the subject (Holzkamp, 2013; Tolman, 1994). The everyday conditions in our lives provide the premises of our grounds for action and effective extension or restriction of individual possibilities lies in the structure of society. One can improve the subjective quality of one’s life by improving the objective conditions and developing one’s agency as a collective power over the individual and general conditions (Holzkamp, 2013; Tolman, 1994). According to critical psychology, learning is a necessary part of a person’s development, agency and conduct in everyday life (Schraube & Marvakis, 2019). People’s conduct in everyday life is constituted by learning, and this learning is related to learners’ intentions, which unfold between subjects and the world. Reflecting on how we actively participate in creating the world and what it means to our subjectivity becomes our foundation for generalization (Busch-Jensen & Schraube, 2019).

Foucault’s (1981) reflections on governmentality, power and discourse have been used to examine the situation of unemployed people. Foucault (1981) sees discourses as central tool for government actions. Discourse serves as a means through which to establish a public consensus regarding the general values and concepts, what is considered normal, etc. (Billmann, 2019). People view their life through certain discursive lenses. Social discourses are presented to us daily in multiple ways and Billmann (2019) argues that it is possible to link Foucault’s concept of discourse and Holzkamp’s reflection on the conduct of everyday life within the reason discourse. Individuals correspond to their ‘subjective’ reasons (Holzkamp, 2013), and this reason discourse implies an inner engagement with social meanings in the form of discourses with which the individual is confronted (Billmann, 2019). Social meanings show up to the individual as opportunities to act. Throughout their everyday lives, people are exposed to masses of discourses to which they must relate one way or another.

In this study, the focus is on societally mediated individual experiences (Holzkamp, 2013). Employability and its development are strongly emphasized in the European Council and European Commission documents from 2001 onwards, and in Finland, discourses concerning descriptions of and solutions to youth unemployment in these documents have been examined by Mertanen (2017). From the political discussion underpinning the Youth Guarantee, she found four discourses based on different conceptions of human beings: early intervention, activation, lifelong learning and employability discourses. In the early intervention discourse, young people are passive objects of measures and need to be directed from one system to another due to apathy and reduced opportunities to obtain employment. In the activation discourse young people are treated not only as objects of guidance but also as active agents. Activation discourse states that social and employment benefits received by young people should be conditional. In the lifelong learning and employability discourses, the world of work is seen as unstable and insecure: young people are offered roles as active developers of their skills and capabilities to improve their employability. The lifelong learning discourse also identifies the need for extra support and guidance, so workshops and apprenticeships are considered essential tools for lifelong learning (Mertanen, 2017).

The data were collected from a foundation in Northern Finland, which provides workshop activities. We explore how participants describe personal transformation borne by workshop participation and whether and how participants’ descriptions were affected by current prevalent discourses on youth (un)employment. In the analysis, we identified (1) how participants assessed their situations; (2) their thoughts, feelings and actions in relation to their situations; and (3) their grounds for those thoughts, feelings and actions. Further, we identify the specific discourses that were characteristic of participants’ grounds for action, utilizing a specific FOG analysis (fabric of grounds) focusing on subjective grounds for action (Suorsa, 2019). After introducing the youth workshop concept and project from which the data were collected, we present the details of the FOG-analysis and its results by describing participants’ experiences in their relation to the discourses.

## Non-formal Learning in Workshops

Workshops as part of Finnish youth work are supposed to improve young people’s skills and abilities so that they might access education and training, complete their studies and become employed (Act of Youth, 2016). Workshop activities are part of the Youth Guarantee, which promises the place of work, internship, work trial, etc. for all under 25 years and under 30 years graduates outside work and learning within 3 months of them becoming unemployed (Ministry of Education & Culture, 2019; TEM, 2019). One of the important aims is to encourage people to be active participants in their own lives rather than merely active and obedient taxpayers, and this goal is achieved through an individual approach and participant orientation (Komonen, 2014). In workshops, systematic processes are tailored to individual needs, with activities, work and learning conducted through practical experience (Bamming & Hilpinen, 2020). The workshops are intended for both young people and adults who find themselves disadvantaged in the labour market. Participants are typically directed to the workshops by the Employment and Economic Development offices and municipal social services. The coaching periods lasted a total of 1–6 months in 2019, and after the workshop period, 80% of the young people moved successfully forward. The workshops operate in Finnish municipalities and are administered by the municipalities in most cases and by associations or foundations in rare cases. Financing of the workshop activities is multi-channelled and includes municipal funding, state aid, other public funding and grants, coaching service revenue and income from workshops selling their products and services (e.g., catering, woodworking, handicrafts, laundry, transportation). There were 26,000 people trained at workshops in 2019. More than half of the trainees were under 29 years old and half of the young trainees had only compulsory school, and one-third of them had a vocational qualification (Bamming & Hilpinen, 2020). The workshops operate at the intersection of youth work, social services, rehabilitation, the education system and the labour market. Despite not being part of the formal education system, in some workshops, it is possible to complete part or all a full vocational qualification (Paving the Way, 2017). Research has suggested that coaching in workshops strengthens young people’s confidence and ability to influence their lives (Wrede-Jäntti, 2018). For participants to be successfully included in workshops, activities should be perceived as meaningful (Mäntyneva & Hiilamo, 2018). When participants can influence their participation, it increases the sense that they are managing their own lives. Workshops are rooted in youth work, which sees young people as valuable and constitutes the self-understanding of many youth work professionals (Nieminen, 2014). Youth work has always been voluntary, guided and communal, and its task is to help young people join society and to offer them learning opportunities for personal, social and cultural development. Youth work is not based on young people’s problems, but on the opportunities that come with receiving the support they need.

The data were collected from a foundation in Northern Finland that provides workshop activities; it also organizes and develops social employment and rehabilitation. The workshops offer the opportunity to work in a variety of jobs such as information and communication technology, cookery, carpentry, textiles, arts, recycling, laundry services, metalwork and transportation. The foundation managed two projects from 2009 to 2019 that provided practice-based learning at the workshops. Skills and competences were compared with the national curricula and could be acquired and recognized at the workshops. For the purposes of recognition and documentation, there was an online application, and skills were always validated by educational institutions. During the projects, approximately 130 workshop participants used the application, which was available on the Internet via computer or mobile phone, to document their skills and competences.

The data primarily consisted of conversations in which ten participants described their situations before and during the period in the workshops with opportunity to study, as well as their hopes for the future. Only one conversation took place with each participant. Participants were between the ages of 24 and 36, half of whom were females and half of whom were males. They were a heterogeneous group that fluctuated between unemployment, employment, studies and clientship with various services (see Aaltonen et al., 2015). Some of them had attended workshops for years, and some were there for the first time. What their situation had in common was that they were not satisfied with their current situation and education but did not see ordinary vocational training as a solution. All ten participants had already been engaged in rehabilitative work activities for some time and were willing to study or look for a job. In these workshops, they aligned their studies with the training, and they had a personal coach who supported them when necessary. The participants were not a specially selected group but instead were regular participants in the 2017–2018 project. The activity discourse affected all participants, as workshop activities were necessary for them to receive social benefits. Participating in the project promoting non-formal learning was voluntary. The participants were informed about the nature and purpose of the research and subsequently gave written consent.

The conversations were conducted using the Abilitator questionnaire from the Finnish Institute of Occupational Health (2019), which is a method of active self-assessment of work and functioning, and the Foundation was testing the questionnaire at the time of the study. The questionnaire offered a uniform approach and acted as a tool for facilitating discussion with participants. The Abilitator questionnaire comprises five sections: general work ability and functional capacity, social functioning, psychological functioning, cognitive functioning, and physical functioning.

The study is also ethnographically informed (Smith, 2001), as the first author of this study has been working at the Foundation for 13 years as an individual trainer, and she was familiar with the interviewees. Audio recordings of participants’ conversations were transcribed and analysed. Primarily, the participants talked about their work, education and aspirations for the future, which were categorized as background information in the questionnaire but provided important information about how participants perceived their situations. The transcript contains 22,595 words.

## FOG-Analysis: Identifying Participants’ Experiences and Their Relation to Relevant Discourses

In the FOG-analysis (Suorsa, 2019), participants’ descriptions were identified: (1) their situations as they described them; (2) their thoughts, feelings and/or actions as they described them; and (3) their grounds for those thoughts, feelings and actions. The researcher reconstructed summaries from participants’ descriptions in which these dimensions were included. The conversations were informal, and the researcher was able to ask for clarification immediately if she did not understand something.

The reconstructed 30 summaries (FOGs) were then systematically scrutinized to identify the discourses in participants’ experiences. We used a timeline to show whether and how participants’ grounds for action changed when their situations changed. In practice, the 30 FOGs were read individually, asking if the rule for discourse X was fulfilled. The rules are presented in Table 1. The result was a table of 30 FOGs with remarks on which discourses were identified in each FOG. At times, discourses were simultaneous and overlapping (see Mertanen, 2017).

**Table 1:** Common discourses in the workshop framework

Discourse	Vulnerability	Lifelong learning/embloyability	Youth work/workshops
Instantiation	Training projects consist of shaping the self to increase employability (Brusila & Ryynänen, 2017)	The participant must show her/his activity by participating in activities such as workshops to receive social and employment benefits (Mertanen, 2017)	Voluntary, guided, and communal, its task is to help young people join society and to offer them learning opportunities for personal, social, and cultural development (Nieminen, 2014)
Ethos	Unemployment is seen as a personal problem, not a structural one; that is, the lack of employment is due to a lack of employability, which can be cured by becoming more willing to adapt. Focus on emotional and psychological vulnerability (Brusila & Ryynänen, 2017)	Prefers young people to be active developers of their skills and capabilities to improve their employability (Mertanen, 2017) Lifelong learning identifies young people’s need for extra support and guidance (Mertanen, 2017)	Sees young people as valuable Focuses on young people’s opportunities rather than problems and gives them the support they need (Nieminen, 2014)

## From Vulnerability Towards an Active Stance?

Participants’ backgrounds were similar in the sense that only two of them had a secondary education, and eight had previously quit their vocational studies one to three times. They all had been unemployed at some point: six of them had never had a paid job, and the rest had only worked occasionally. They described their current situations as consisting of non-formal studies combined with workshop activities. Participants generally expressed a desire to finish school and find jobs, except two participants who wanted to continue the workshops. Table 2 shows the participants’ activities (“I quit school”) together with the grounds for action they offered (“…because I was afraid of social situations”). The activities and grounds are categorized according to discourses (Table 1) that were identified in the grounds.

**Table 2:** Participants’ grounds for action and their relationship to the discourses concerning their situation

Discourses Participants’ grounds	Vulnerability	Lifelong learning/youth work	Youth work/employability
Before the intervention	I quit school because: • I was so down. • I had difficulties to concentrate. • I am afraid of social situations. • I was young and stupid. • I failed exams three times. • I was slow.	I quit school because: • I never got the help I needed. • I was promised my own curriculum, but it never happened. I don’t have a job because: • I don’t have any qualifications. • Everyday difficulties have slowed down employment.	
During the intervention	I am participating in non-formal learning in workshops because: • it was a waste of time, but I still got money. Everyone praised me, but it was just talk. I know nothing.	I am participating in non-formal learning in workshops because: • It is possible to take breaks. • I got help when I needed it. • I could ask questions hundreds of times. • I found training with the help of the coach. • I got a certificate from the school. • It has been easy to study. • I got the feeling I am suitable.	
After the intervention	I continue with the workshops because: • I might study again, but the threshold for getting back to school is too high. I did so badly last time. I would not be able to do anything anyway. • I would get less money if had a paid job.	I am about to finish my studies because: • I got a lot of support and strength from the program. • I have illnesses but they don’t matter; I must continue studying.	I am looking for a job because: • I don’t have to lay in bed at home. • I don’t have to sit at home as I have for two years. • I have been unemployed all my life. • I have hope now.

Participants often described their path to the foundation as resulting from their own failures (vulnerability, individualization). Participants represented themselves as passive objects and thus did not act on opportunities to improve their situations:

“I dropped out of vocational school. I could not finish it, because I was so down”.

“I have dropped out of vocational studies three times. I was not interested in them; I was young and stupid.”

Three of the participants indicated external grounds for action. One of them told that he saw reasons for his failure in his difficulties concentrating and circumstances. He could have received support in his upper secondary school, but in the Finnish system, it is not automatically provided in vocational studies (see Eriksson, 2018):

“I almost had my qualification. But I could not do the final test because it is difficult to concentrate. I also feel that nobody helped me at school.”

Two participants explained that they were unemployed because of family issues; one needed a job, and the other needed qualifications.

“I have been unemployed for many years, taking care of my family. I have done two vocational exams. Everyday difficulties have slowed down employment opportunities a bit.”

“I have been unemployed for three years; I have been a house mom. I have not done any vocational examinations yet. I am frustrated. I have time now, but I do not have a job because I do not have any qualifications.”

After participating in the project, participants talked about themselves more as active subjects. They discovered more flexible and tailored ways of working and learning, such as the possibility of working part-time. They also felt more motivated and received support when needed. When focusing on the individual support that is needed, there is the possibility of avoiding social stigma, which might be associated with the diagnosis (see Eriksson, 2018). This could be an exemplification of what Nieminen (2014) proposed: ethos of youth work as a counterforce to the categorizing and labelling of young people. Youth work does not focus on young people’s problems but rather on creating opportunities for them when they obtain appropriate support. Participants gained new skills and competences through learning by doing. They learned to recognize their own strengths through work and feedback. The discourse of vulnerability and individualisation seemed to fade as the lifelong learning discourse began to take its place for participants who had benefited from positive experiences and tailored support:

“I do not find it so stressful when I am working and studying only three days a week and taking breaks.”

“I graduated. I am satisfied with myself, because I found it easier to study when I could ask a question hundreds of times without anybody getting mad, and I got the help I needed.”

“The project worker helped me to get training in an enterprise. Her contacts played a significant role. Then, she helped me to find education; I could not find them on my own, but now I got the feeling that I was suitable. I got a certificate for my studies from the school.”

When participants described their hopes for the future, their grounds for action centred on their desires to avoid returning to their former situations. Lifelong learning and employability discourses emerged when participants wanted to study more and obtain jobs. Workshop activities and non-formal learning seem to have improved participants’ skills and abilities, as they subsequently felt as though they had gained access to education and employment:

“I am quite satisfied with my life now. And I am going to be even more satisfied when I am qualified and get a job. I have been unemployed and trying to study for so many years.”

Only one participant mentioned his illness, but he thought that he must study, nevertheless:

“I am wondering about my situation. I am quite sure I am going to handle it, and I think I am going to be able to study even though I have illnesses.”

There were two participants who wanted to stay in the workshop environment. In one case, the participant’s grounds changed from being afraid of social situations and dropping out of school to wanting to stay in a programme with unemployment benefits:

“I am not looking for a job. I do not think I am able to do a full-time job, and I think I would lose some benefits if I took a paid job. I want to continue working in the programme.”

## Discussion

When Mertanen (2017) identified discourses in European Union policy programmes relating to youth unemployment, she found that these discourses assigned young people both active and passive roles. Discourses linked to vulnerability and individualisation (Mertanen’s ‘early intervention’ discourse) assigned participants a passive role when explaining why they failed at school. Prior to the study, participants labelled themselves ‘inappropriate’ for the school because of their vulnerabilities. (see Brunila et al., 2018; Furlong et al., 2011; Thompson, 2011) As Brunila et al. (2018) states, once young people are categorised as vulnerable, they tend to learn how to belong to that category and the policies of support systems can be repeated endlessly as the problem is always to be found in the young person.

Initially, participants experienced restricted opportunities because of their vulnerabilities, but ultimately, they saw new opportunities and developed the motivation to avoid their former situations. The main changes in motivation seem to have occurred during the project. The participants developed their agency as a means of overcoming individual life conditions (Holzkamp, 2013). Youth work ethos in the workshop environment, which sees young people as individuals with personal potential, seems to support young people in initiating change (see European Union, 2020; Komonen, 2014; Nieminen, 2014). It seems that the participants’ scope of possibilities expanded during the projects. Participants took an active stance and were motivated to change their situations for the better. Participants’ experiences also illustrate the general ideas of agency and learning, according to which, e.g. the difficulties in learning are acceptable—without being paralyzing—when participants see the learning situations as possibilities to enhance the quality of their lives (e.g. Holzkamp, 2013; Schraube & Marvakis, 2019).

In the lifelong learning discourse, participants described their roles as more active, first by blaming school for failures and then by attributing new study methods to participation in workshop activities. Lifelong learning discourse seemed to strengthen participants’ perceptions of their opportunities to develop their abilities and contribute to society (Miettinen, 2016). When discussing their hopes for the future, participants mentioned a desire to avoid their former passive roles and unemployment (see also Haikkola & Myllyniemi, 2020). The results of this research support the results of Mäntyneva and Hiilamo (2018), which indicate that workshop activities increase the sense that one is managing one’s own life. They added that a positive future horizon and supporting activities, such as non-formal learning, reinforce a sense of involvement. There seems to be a need for individualised services and perceptions of young people as unique individuals who have resources and capabilities for coping despite their symptoms, disabilities or illnesses (Aaltonen et al., 2015). If social security systems are more flexible and stimulating, perhaps they could encourage particularly low-skilled young people to continue their studies and invest in their own human capital (Hiilamo et al., 2017). Lifelong learning gives opportunities to live meaningful lives, but should it be more rooted in individual needs than in the needs of the market? The research conducted in the workshops shows that if participation in training is voluntary, it supports participants’ autonomy. Conversely, self-determination might be adversely affected if participation is compulsory (Mäntyneva & Hiilamo, 2018).

In our study, when considering common discourses in the workshop framework, participants’ grounds for maintaining and/or changing their life conditions changed. Participation in the project gave participants a new structural environment of support and tailored services. Discourses relating to participants’ grounds for action illuminated the subjective experiences associated with objective circumstances and structures. As a concept and research tool, the FOG seeks to articulate the connection between subjective reasons for action and societal conditions by showing why, under these conditions, it is ‘subjectively functional’ to act and experience in this way. A subjective situation is an assessment of an objective environment, but it is also an assessment of a person’s relationship to the environment and to the possibilities that are effectively available (Holzkamp, 2013; Tolman, 1994).

One of the limitations of this study was that interpretations were not shown to participants, so the subject-scientific process was not completed in this study (Holzkamp, 2013; Suorsa, 2019). In their current form, the FOGs and their relations to discourse are still relatively unrefined. We suggest that continuing the conversations about the discourses with the participants would constitute valuable research and would benefit the participants. This would also correspond to the subject-scientific ideal of conducting the research in collaboration with the participants (instead of seeing them as “objects of research”) and helping them grasp their current possibilities for action (see Holzkamp, 2013). In addition to helping research, this could also be beneficial for clients’ personal growth (e.g., Suorsa, 2015).

Methodologically, we found a subject-scientific approach suitable for this research, as it enables researchers to focus on both structural and individual dimensions of action and experience. The approach is not currently very widely known, but it holds great promise for studying the experiences and practices of supporting young people. In addition to studying clients’ and students’ perspectives (e.g., Dreier, 2008; Juhl, 2019; Peltola & Suorsa, 2020), it is also necessary to use this approach to understand professionals’ perspectives and their relations to their working conditions (e.g., Peltola et al., 2020; Raetsaari et al., 2020).

A further theme for research would be to go deeper into the subjective functionality of the given discourses to participants in their trajectories of everyday living. Another interesting topic for further study could be to examine the—intended and unintended—meanings of new learning technologies for personal transformations in workshops. Would there be fewer failures if more flexible study methods were available?

## Conclusion

The workshops in Northern Finland provided young unemployed people with an opportunity to engage in non-formal learning. Skills and competences were compared with those specified in national curricula and could be acquired at the workshops and validated by an educational institution.

The focus of our study was to examine how participants described personal transformation borne by workshop participation and whether and how participants’ descriptions were affected by current prevalent discourses on youth (un)employment.

The findings showed similarities in participants’ descriptions of their situations prior to participation in the workshops. In these descriptions, a vulnerability discourse was identified. There were also similarities in participants’ descriptions of the meaning of workshop activities. Clients saw personalized support, attention and patience as the most essential qualities of workshop supervisors. Finally, we identified similarities (and some differences) in participants’ descriptions of their current situations and their hopes for the future. Their descriptions were characterized by prevalent discourses of lifelong learning, youth work and employability.

The findings suggest that workshops can be—from the participants’ perspectives—an effective way of helping young unemployed people find their place in society. Based on our research, we suggest that, in supporting them, attention should be given to participants’ experiences and to how discourses affect the way young people see themselves and their possibilities for action.
